# Evalutaion of two different control charts (I and U) in the study of mutiresistant bacteria contact precautions dynamics in a non-endemicity hospital setting

**DOI:** 10.1186/1753-6561-5-S6-P11

**Published:** 2011-06-29

**Authors:** G Mestre, C Berbel, P Tortajada, JR Agüera, L Cort, J Zaragoza, JA Martinez, J Rodriguez-Baño

**Affiliations:** 1 Nosocomial Infection Control Unit, Barcelona, Spain; 2 Microbiologic Unit, Delfos Medical Centre, Barcelona, Spain; 3 Infectious diseases Unit, Hospital Clinic, Barcelona, Spain; 4 Infectious diseases and Microbiology Unit, University Hospital Virgen de Macarena, Sevilla, Spain

## Introduction / objectives

Data about usefulness of Statistical Process Control in the study of contact precautions (CP) dynamics for control of resistant bacteria are scarce.

## Methods

Retrospective cohort study. All admitted patients colonized or infected by MRSA, MDR-PAE and Abaumannii from 2005 to 2010 were included. Period I (01/2005-04/2008) without active surveillance; Period II (05/2008-12/2010), active surveillance in all patients admitted to the Intensive Care Unit and in readmitted previously colonized patients. Clonality was studied by PFGE. Charts: I-graph (Y axis shows the days between two consecutive CP;X axis: consecutive number of CP) and U-graph (CP per 10.000 patients days clustered by quarters).

## Results

The average days between two consecutive CP in period I and period II were: 20 vs 31 days for MRSA, 41 vs 46 days for MDR-PAE and 53 vs 59 days for Ab, respectively. The average rate of patients under CP per 10.000 patient-days were: 3.19 vs 2.51 for MRSA, 1.40 vs 1.49 for PAE MR and 1.35 vs 1.09 for Ab (period I and II respectively). All outbreaks were coincident for special negative causes in graph I while the U graph only detected 2 out of 5 (type II error). All special positive causes was detected by graph I just after outbreak intervention (more days between consecutive CP). Although graph I often showed positive special causes in 5 out of 12, a type I error could not be ruled out.

## Conclusion

In a non endemic setting when few events are present, the I graph is more sensitive and less specific, while the U graph is more specific but less sensitive.

## Disclosure of interest

None declared.

